# A New Temperature Correction Method for NaI(Tl) Detectors Based on Pulse Deconvolution

**DOI:** 10.3390/s23115083

**Published:** 2023-05-26

**Authors:** Jianming Xie, Liu Yang, Jinglun Li, Sheng Qi, Wenzhuo Chen, Hang Xu, Wuyun Xiao

**Affiliations:** State Key Laboratory of NBC Protection for Civilian, Beijing 102205, China; xjm198405@163.com (J.X.); yangliu@sklnbcpc.cn (L.Y.); dmcaspectra@126.com (J.L.); qscups@163.com (S.Q.); voldemort@yeah.net (W.C.); xu_hang0605@163.com (H.X.)

**Keywords:** NaI(Tl) detector, temperature correction, pulse deconvolution, trapezoidal shaping

## Abstract

To overcome the temperature effect of NaI(Tl) detectors for energy spectrometry without additional hardware, a new correction method was put forward based on pulse deconvolution, trapezoidal shaping and amplitude correction, named DTSAC. To verify this method, actual pulses from a NaI(Tl)-PMT detector were measured at various temperatures from −20 °C to 50 °C. Pulse processing and spectrum synthesis showed that the position drift of the ^137^Cs 662 keV peak was less than 3 keV, and the corresponding resolution at 662 keV of the sum spectra ranged from 6.91% to 10.60% with the trapezoidal width set from 1000 ns to 100 ns. The DTSAC method corrects the temperature effect via pulse processing, and needs no reference peak, reference spectrum or additional circuits. The method solves the problem of correction of pulse shape and pulse amplitude at the same time, and can be used even at a high counting rate.

## 1. Introduction

NaI(Tl) detectors are the most widely used scintillation detectors due to their low price and good reliability [1]. However, their radiation response has a remarkable temperature effect [2,3,4], leading to unacceptable spectrum drifts in field applications with varying temperatures. In addition, a long luminescence decay time at low temperatures [2,3,4] makes them unfit for high count rate applications. Temperature correction for NaI(Tl) detectors has generally been performed using three types of spectra stabilization methods. The first is to stabilize the position of some reference peaks by adjusting the high voltage of the photomultiplier tube (PMT) [5] or the gain of the amplifier [6,7]. The second is to continuously correct the spectra in time series [8,9,10] based on the reference peak [5,11] or the reference spectrum [8,9]. The last is to use a temperature-dependent correcting factor to correct the amplitude of each pulse [12]. The use of a reference peak or reference spectrum requires notable peaks or a stable radiation field, and this requirement is not always satisfied. Adjustment of the high voltage or amplifier gain increases the complexity of the electronic system. The last method is attractive and has been applied in the commercial instrument identiFINDER [12]. However, none of the traditional methods can solve the difficult problem of the application of a high count rate due to the long decay time at low temperatures.

In this paper, a new temperature correction method based on pulse processing is put forward to solve the problem of correction of pulse shape and pulse amplitude at the same time.

## 2. Materials and Methods

### 2.1. Method Description

**Figure 1:**
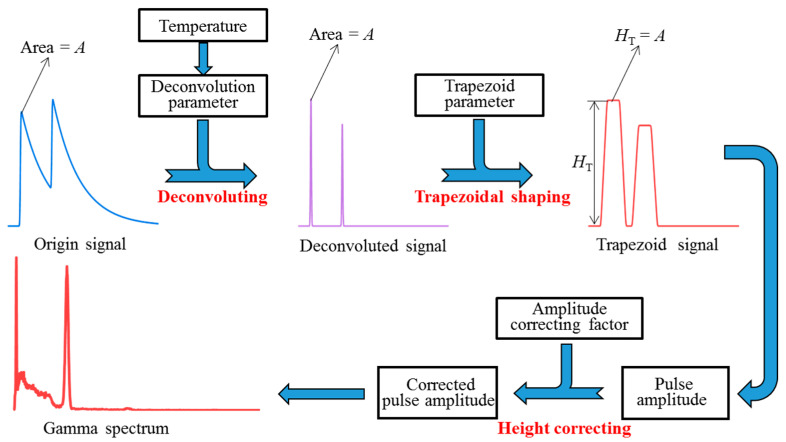
Schematic diagram of the proposed method.

For the same energy deposition of the incident gamma ray, both the decay time and the area of the pulses from the NaI(Tl) and PMT system vary with the temperature. In order to correct the two effects, a three-step pulse processing procedure using the DTSAC method was designed, namely deconvolution [13,14], trapezoidal shaping [15], and amplitude correction [12], as shown in Figure 1. The first two steps correct the effect of temperature on pulse duration, and the latter step corrects the effect of temperature on pulse area. A uniform pulse model with different parameters was used to describe the NaI(Tl) pulses at different temperatures. In the deconvolution step, all pulses at different temperatures were deconvolved into quasi-δ pulses with an invariant area. In the trapezoidal shaping step, quasi-δ pulses were shaped into trapezoidal pulses, ensuring that each amplitude was equal to the original pulse area. In the amplitude correction step, the amplitude values extracted from the trapezoidal pulses were corrected to be equal to those at the reference temperature according to a previously calibrated temperature response function.

#### 2.1.1. Pulse Model and Deconvolution

The convolution pulse model in the time domain proposed in our previous work [14,16] is used to describe the PMT anode pulse of the NaI(Tl) detectors, and it is rewritten as

(1) $$\begin{array}{ll} f(t) & =A\cdot \frac{1}{\sqrt{2\pi }\sigma }e^{-t^{2}/2\sigma^{2}}*\left(\sum_{i=1}^{M}\frac{\phi_{i}}{\tau_{i}}\cdot \left(e^{-t/\tau_{i}}u(t)\right)\right)*\left(\sum_{j=1}^{N}\frac{\varphi_{j}}{\lambda_{j}}\cdot \left(e^{-t/\lambda_{j}}u(t)\right)\right),\\ & =A\cdot f_{1}(t)*f_{2}(t)*f_{3}(t) \end{array}$$

where the asterisk (*) is a convolution operator, *u*(*t*) is the unit step function, *A* is the area of the pulse, *σ* is the standard deviation of the Gaussian function, *M* and *N* are the number of exponential components, *τ_i_* and *λ_j_* are the decay constants of the exponential functions, and *φ_i_* and *ϕ_j_* are the proportions of each exponential components (calculated by area), which satisfy

(2) $$\sum_{i=1}^{M}\phi_{i}=1,\sum_{j=1}^{N}\varphi_{j}=1.$$

In this model, the anode pulse signal is described as a convolution of three functions. The first one, *f*_1_(*t*), is a Gaussian function. The other two, *f*_2_(*t*) and *f*_3_(*t*), are the sum of several exponential functions. *f*_1_(*t*), *f*_2_(*t*) and *f*_3_(*t*) are all normalized functions of area instead of amplitude. The commonly used uni-exponential model, bi-exponential model, Gauss-exponent convolution model and others can be regarded as special versions of this model. Due to the influence of temperature, even for the same energy deposition, the parameters *A*, *σ*, *τ_i_*, *λ_j_*,*φ_i_* and *ϕ_j_* are all slowly varying functions of temperature, *T*. Model parameters at specific temperature can be obtained by fitting the average pulse. The relationship between model parameters and temperature can be established via function fitting or interpolation.

The *z* transformation of *f*(*t*) is

(3) $$\begin{array}{ll} F(z) & =A\cdot F_{1}(z)\cdot F_{2}(z)\cdot F_{3}(z)\\ & =A\cdot F_{1}(z)\cdot \left(\sum_{i=1}^{M}\frac{\phi_{i}}{\tau_{i}}\cdot \frac{1}{1-d_{i}z^{-1}}\right)\cdot \left(\sum_{j=1}^{N}\frac{\varphi_{j}}{\lambda_{j}}\cdot \frac{1}{1-c_{j}z^{-1}}\right), \end{array}$$

where

(4) $$d_{i}=e^{-T_{s}/\tau_{i}},c_{i}=e^{-T_{s}/\lambda_{j}}.$$

*T*_s_ is the sampling period. As developed in in our previous work [16], the original pulse, *f*(*t*), can be deconvolved into a narrow Gaussian pulse, *A*∙*f*_1_(*t*), with an invariant area, and its *z*-domain expression is

(5) $$A\cdot F_{1}(z)=D(z)\cdot F(z)=F_{2}^{-1}(z)\cdot F_{{}_{3}}^{-1}(z)\cdot F(z),$$

where

(6) $$\begin{array}{cc} D(z) & ={\left(\sum_{i=1}^{M}\frac{\phi_{i}}{\tau_{i}}\cdot \frac{1}{1-d_{i}z^{-1}}\right)}^{-1}\cdot {\left(\sum_{j=1}^{N}\frac{\varphi_{j}}{\lambda_{j}}\cdot \frac{1}{1-c_{j}z^{-1}}\right)}^{-1}\\ & =\frac{\prod_{i=1}^{M}(1-d_{i}z^{-1})\prod_{j=1}^{N}\left(1-c_{j}z^{-1}\right)}{\sum_{k=0}^{M+N-2}a_{k}z^{-k}} \end{array}$$

*D*(*z*) is the deconvolution filter, and depends on the temperature, *T*. *a_k_* is the polynomial coefficient determined only by model parameters *τ_i_*, *λ_j_*, *φ_i_* and *ϕ_j_*. When *M* = 2, *N* = 1, *δ* is very close to 0, and a simplified model such as (7) can be obtained. It is the convolution between an exponent function and the sum of two exponent functions.

(7) $$\begin{array}{ll} f(t) & =\frac{A}{\tau_{0}}e^{-t/\tau_{0}}u(t)*\left(\frac{\phi }{\tau_{1}}e^{-t/\tau_{1}}u(t)+\frac{1-\phi }{\tau_{2}}e^{-t/\tau_{2}}u(t)\right)\\ & =\frac{A\phi }{\tau_{1}-\tau_{0}}(e^{-t/\tau_{1}}-e^{-t/\tau_{0}})+\frac{A(1-\phi )}{\tau_{2}-\tau_{0}}(e^{-t/\tau_{2}}-e^{-t/\tau_{0}}) \end{array}$$

In Equation (7), *A* is the pulse area, *τ*_0_ is the rising time constant, *τ*_1_ and *τ*_2_ represent the time constant of the fast component and the slow component, respectively, and *φ* is the proportion of the fast component.

The *z*-domain expression of the deconvolution filter *D*(z) is

(8) $$D(z)=\frac{1}{F(z)}=\frac{\left(1-d_{0}z^{-1})(1-d_{1}z^{-1})(1-d_{2}z^{-1}\right)}{\left(\eta_{1}d_{1}+\eta_{2}d_{2}-(\eta_{1}+\eta_{2})d_{0}\right)z^{-1}+\left(\eta_{1}d_{0}d_{2}+\eta_{2}d_{0}d_{2}-(\eta_{1}+\eta_{2})d_{1}d_{2}\right)z^{-2}}.$$

Ignoring the one-period delay of the deconvolution result, the time domain algorithm can be expressed as

(9) $$V_{O}(n)=\frac{1}{\eta_{1}d_{1}+\eta_{2}d_{2}-(\eta_{1}+\eta_{2})d_{0}}\left(V_{I}(n)-b_{1}V_{I}(n-1)+b_{2}V_{I}(n-2)-b_{3}V_{I}(n-3)-b_{4}V_{O}(n-1)\right),$$

where

(10) $$\begin{array}{l} \eta_{1}=\frac{\phi }{\tau_{1}-\tau_{0}}\\ \eta_{2}=\frac{1-\phi }{\tau_{2}-\tau_{0}}\\ b_{1}=d_{0}+d_{1}+d_{2}\\ b_{2}=d_{0}d_{1}+d_{0}d_{2}+d_{1}d_{2}\\ b_{3}=d_{0}d_{1}d_{2}\\ b_{4}=\eta_{1}d_{0}d_{2}+\eta_{2}d_{0}d_{1}-(\eta_{1}+\eta_{2})d_{1}d_{2} \end{array}$$

#### 2.1.2. Trapezoidal Shaping and Amplitude Correction

In the actual situation, the pulse deconvolution operation can cause two problems. One is the signal-to-noise ratio (SNR) decreasing remarkably; the other is that the deconvolution pulses have different widths because the parameter *σ* depends on temperature, *T*. In order to improve the SNR, the trapezoidal filter is used to shape the deconvolution pulse, the *z*-domain expression of which is

(11) $$H(z)=\frac{\left(1-z^{-n_{a}})(1-z^{-n_{b}}\right)}{n_{a}{\left(1-z^{-1}\right)}^{2}},$$

where *n*_a_ and *n*_b_ are trapezoidal parameters, and *t*_Top_ = (*n*_b_ − *n*_a_)*T*_s_ and *t*_Width_ = (*n*_b_ + *n*_a_)*T*_s_ are the time width of the flat-top and bottom, respectively. In order to avoid a ballistic deficit caused by *σ*(*T*), it is required that *t*_Top_ > 6*σ*_max_, which can be easily met since *σ* is generally about 10 ns. Trapezoidal parameters can be selected according to the pulse count rate to achieve the balance between pulse throughput and energy resolution.

If the actual pulse is exactly consistent with the pulse model, the amplitude, *H*_T_, of the trapezoidal pulse will be equal to the original pulse area, *A*. Considering the inaccuracy of the pulse model, the model error correction factor, *κ*, is introduced to make *κH*_T_ = *A*, and *κ* is a function of temperature and trapezoidal parameters, denoted as *κ*(*T*,*n*_a_,*n*_b_). The more accurate the model is, the closer to 1 the value of *κ* would be. The area, *A*, of the original pulse for γ-rays with specific energy is mainly determined by the light yield of the scintillator and the PMT gain, both of which are affected by temperature [12]. The area of the original pulse at a temperature, *T*, is denoted as *A*(*T*), and the one at reference temperature, *T*_0_, is denoted as *A*_0_. Equation (12) can be obtained while introducing the area correction factor *ε*(*T*) = *A*_0_/*A*(*T*).

(12) $$\varepsilon (T)A(T)=\varepsilon (T)\kappa (T,n_{a},n_{b})H_{T}(T)=\varepsilon^{\prime }(T,n_{a},n_{b})H_{T}(T)=A_{0},$$

*ε*′(*T*,*n*_a_,*n*_b_) = *ε*(*T*)*κ*(*T*,*n*_a_,*n*_b_) is the amplitude correction factor. The corrected trapezoidal height is equal to the area of the original pulse at the reference temperature, which is independent of temperature, so as to achieve spectrum stabilization. The correction result calculated by *ε* has some deviation due to the model error, while the one calculated by *ε*′ is more accurate. However, *ε* is easy to use, since it is independent of trapezoidal parameters, while *ε*′ should be calculated according to trapezoidal parameters.

### 2.2. Pulse Data Acquisition and Preprocessing

A Φ5.08 cm × 5.08 cm NaI(Tl) detector coupled to Hamamatsu CR173-01 PMT [17] was used, and a 1 μCi ^137^Cs point source was located near to its front end. The experiments were carried out in a KOWINTEST KW-TH-100X-PC thermostatic chamber (Guangdong Kewen Test Equipment Co., Dongwan, Guangdong, China; temperature control range: −60~150 °C; temperature control accuracy: ±0.5 °C). The pulses were collected at several temperature points from −20 °C to 50 °C with a step of 10 °C. The temperature changing rate was controlled within 5 °C/h in order to protect the NaI(Tl) crystal. Each temperature point was kept constant for 8 h to ensure thermal balance, and the pulses were collected in the last 30 min using a CAEN N6730 digitizer [18]. The pulse data recording length was 8 μs and 1 μs before triggering was reserved as its baseline. The baseline of each pulse was estimated and then subtracted from the recorded pulse. After discarding the data with pulses truncated by the digitizer because of the excessive amplitude and the data with multiple pulses within the acquisition time, 200,000 single pulses were obtained at each temperature point. Pulses from the 662 keV photoelectric peak were picked out according to the pulse area.

## 3. Results

### 3.1. Pulse Model Parameter Fit Result

**Figure 2:**
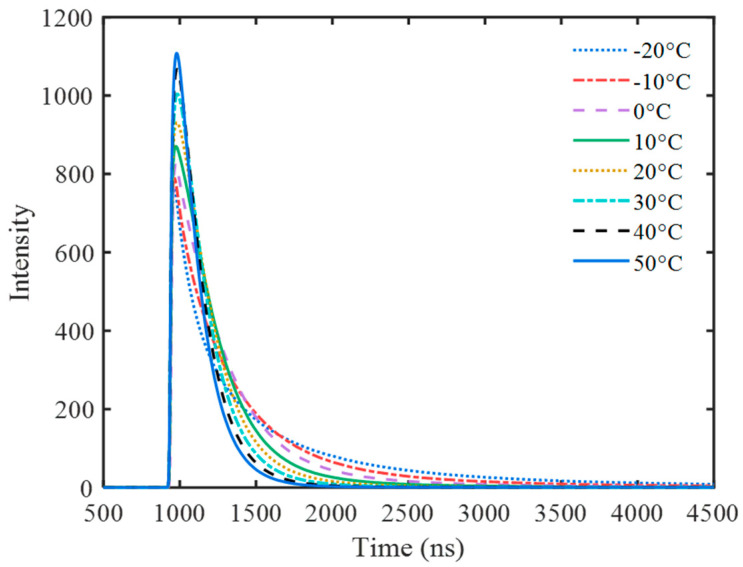
Average pulse of 662 keV at different temperatures.

The selected pulses were aligned and used to calculate the average pulse at each temperature (Figure 2). The results show that the luminescence decay time of the NaI(Tl) scintillators decreases with the increase in temperature, which is consistent with the results in the literature [2,3,4].

**Figure 3:**
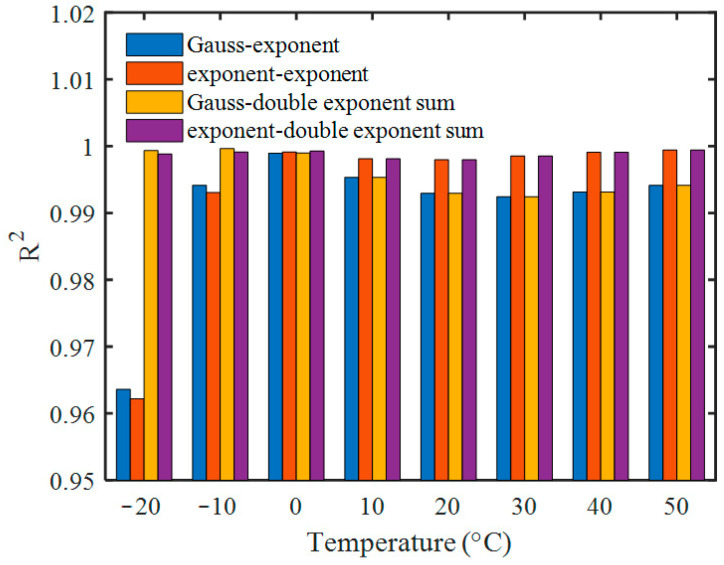
Comparison of R^2^ values of average pulse fitted by different models.

The pulse model in Section 2.1.1 has too many parameters to fit, so the number of parameters should be appropriately reduced to facilitate fitting. Some special cases including the Gauss-exponent convolution model, exponent-exponent convolution model (bi-exponential model), Gauss-double exponent sum convolution model, and exponent-double exponent sum convolution model were used to fit the average pulses at different temperatures. Considering the pulse arrival time *t*_0_, the pulse model function *f*(t) should be modified to *f*(*t* − *t*_0_), where *t*_0_ is also treated as a fit parameter. In order to make the area of the deconvolution pulse equal to that of the original pulse, the pulse area parameter, *A*, was set as the value of average pulse area, which means that *A* does not need to be determined by fitting. The fit results of the first two models were not sensitive to initial values due to the few fit parameters, so the Levenberg–Marquardt algorithm with roughly selected initial values was used for fitting. The fit results of the last two models were sensitive to the initial values due to there being too many fit parameters. An iterative process of alternating optimization was used, and the fit results of the first two models were adopted as the initial values. The R^2^ values fitted by each model are shown in Figure 3. It can be seen that the exponent-double exponent sum convolution model is the best, with its R^2^ values ranging from 0.9980 to 0.9994. The fit parameters are shown in Table 1.

### 3.2. Temperature Correction Result

**Figure 4:**
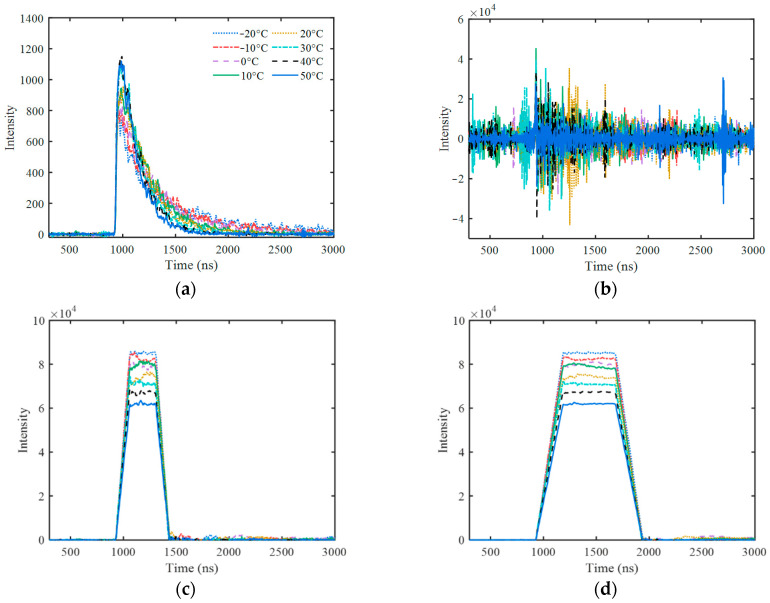
Actual pulses from 662 keV at different temperatures and their processing results. (**a**) Original pulse; (**b**) deconvolution pulse; (**c**) trapezoidal pulse (*t*_Width_ = 200 ns, *t*_Top_ = 100 ns); (**d**) trapezoidal pulse (*t*_Width_ = 1000 ns, *t*_Top_ = 500 ns).

According to different temperatures, the actual pulses from 662 keV are shown in Figure 4, compared with their deconvolution results, and their further trapezoidal shaping results with different parameters. It looks difficult to identify the quasi-δ pulse in the deconvolution signal because of the low SNR. After shaping, the trapezoidal pulses are very obvious. All of the trapezoids have almost the same shape only with different amplitudes caused by different temperatures. The random fluctuation of the trapezoidal flat-top decreases with the increase in the pulse width, *t*_Width_.

**Figure 5:**
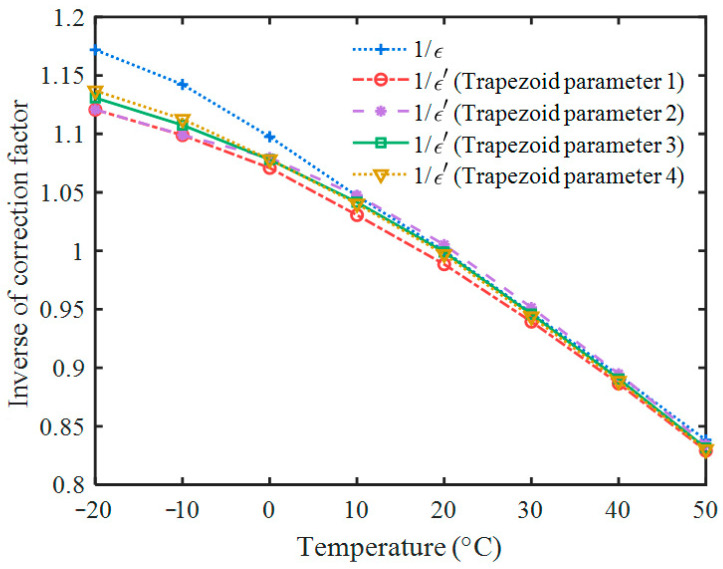
Amplitude correction factors at different temperatures. Trapezoid parameter 1: *t*_Width_ = 500 ns, *t*_Top_ = 100 ns; trapezoid parameter 2: *t*_Width_ = 500 ns, *t*_Top_ = 250 ns; trapezoid parameter 3: *t*_Width_ = 1000 ns, *t*_Top_ = 200 ns; trapezoid parameter 4: *t*_Width_ = 1000 ns, *t*_Top_ = 500 ns.

As shown in Figure 4d, the amplitude values of all trapezoidal pulses extracted at the center of their flat-top were corrected by *ε* and *ε*′ separately. The average areas of 662 keV pulses at different temperatures were used to calculate *ε*, and the average amplitude of the corresponding trapezoidal pulses were used to calculate *ε*′. The relationship between 1/*ε* (1/*ε*′) and temperature are shown in Figure 5.

The quadratic function,

(13) $$1/\varepsilon (T)=a_{0}T^{2}+a_{1}T+a_{2},$$

was used to fit the relationship between *T* and 1/*ε* (1/*ε*′). The fit results of *ε* and *ε*′ for different trapezoidal parameters are shown in Table 2.

As shown in Table 2, the relationships between *T* and 1/*ε*(1/*ε*′) are in good agreement with the quadratic function, and the R^2^ values are all above 0.9993.

**Figure 6:**
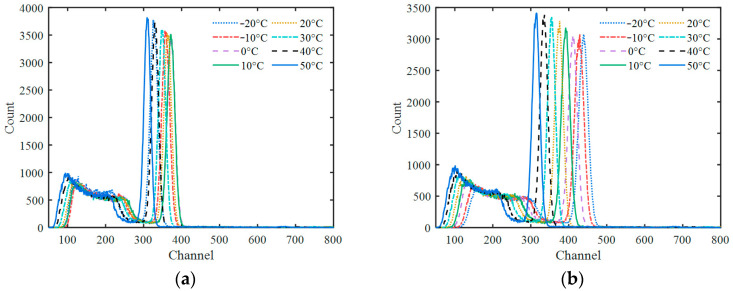

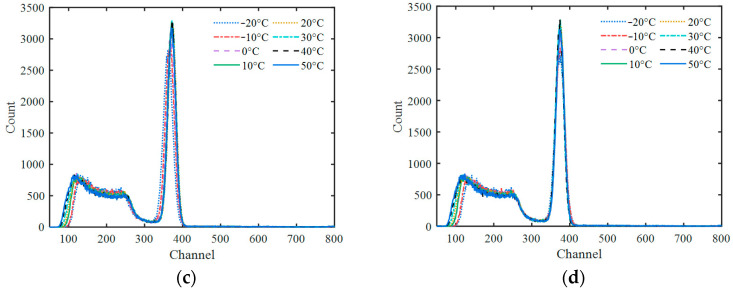
Spectra obtained with different methods at different temperatures. (**a**) Gated integration at 1000 ns; (**b**) gated integration at 7000 ns; (**c**) the proposed method (*t*_Width_ = 1000 ns, *t*_Top_ = 500 ns, *ε*); (**d**) the proposed method (*t*_Width_ = 1000 ns, *t*_Top_ = 500 ns, *ε*′).

All pulse amplitudes corrected by *ε* and *ε*′ calculated by the fitted quadratic function at each temperature were counted and sorted into a spectrum of 1000 channels with a proper channel width. As a comparison, gated integrations with different integration periods from 1000 ns to 7000 ns were performed on the actual pulses, and the amplitude results were converted into spectra in the same way. The gamma spectra at different temperatures obtained using the four processing methods are shown in Figure 6. As can be seen, the spectra obtained via gated integration have obvious spectrum drifts at different temperatures, and the spectrum drift of 7000 ns is more obvious than that of 1000 ns. That is because the pulse area decreases with the increase in temperature, and the luminescence decay time decreases with the increase in temperature. The increase in the total area and the incomplete integration counteract each other to a certain extent when the gated integration time is shorter. However, as seen in Figure 6c,d, after correction with the proposed method, the variation in the peak positions of different temperatures is controlled within a very small range. Especially for the last one, the peak position seems almost independent of temperature when the amplitude correction factor *ε*′ is used.

The overall drifting ranges of the 662 keV peak position of the four methods are 62.9, 124.9, 11.2 and 1.4 channels, respectively. Because the trigger threshold for pulse acquisition was a bit high, the X-ray peaks of 32 keV were removed from the spectra.

**Figure 7:**
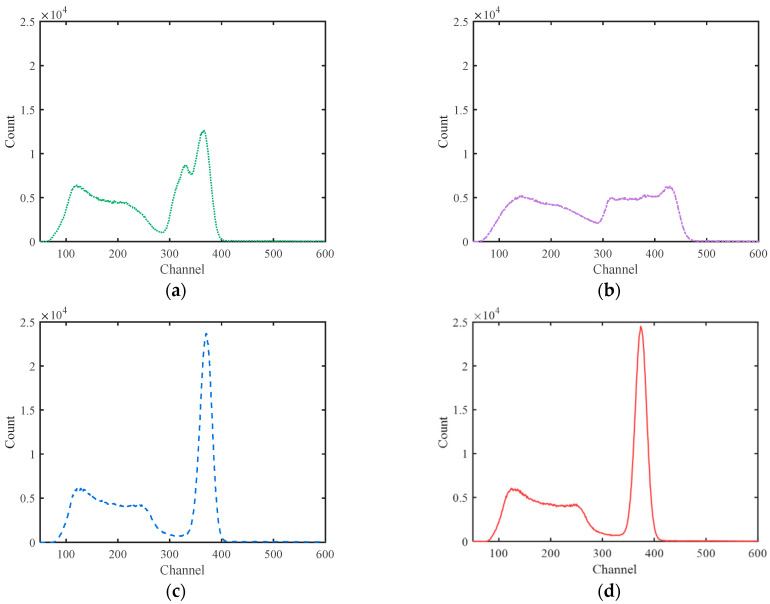
Sum spectra obtained via different processing methods. (**a**) Gated integration at 1000 ns; (**b**) gated integration at 7000 ns; (**c**) the proposed method (*t*_Width_ = 1000 ns, *t*_Top_ = 500 ns, *ε*); (**d**) the proposed method (*t*_Width_ = 1000 ns, *t*_Top_ = 500 ns, *ε*′).

Figure 7 shows the sum spectra added together with the spectra of different temperatures. It can be seen that the two sum spectra obtained via gated integration methods are distorted significantly. However, the other two sum spectra obtained with the proposed method are satisfying. The shape of the 662 keV peak looks very similar to Gaussian distribution, with calculated values of energy resolution being 7.23% and 6.91% for *ε* and *ε*′, respectively. With temperature ranging from −20 °C to 50 °C, the lowest position drift of the main peak was less than 3 keV. This means that the temperature effect of the NaI(Tl) detector was removed accurately.

**Figure 8:**
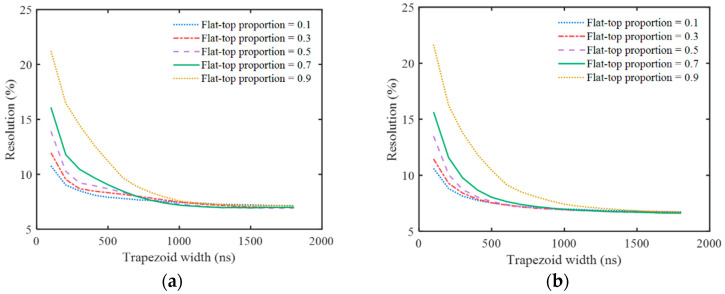
The 662 keV resolution of sum spectra with different correction factors and trapezoidal parameters. (**a**) Amplitude corrected by *ε*; (**b**) amplitude corrected by *ε*′.

In order to study the influence of different trapezoidal parameters on the energy resolution of the sum spectra, different parameters with *t*_Width_ = 100~1800 ns and a flat-top ratio of 0~0.9 were used for trapezoidal shaping. The energy resolutions of the 662 keV peak are shown in Figure 8.

It can be seen that the energy resolution deteriorates with the decrease in *t*_Width_. However, even if *t*_Width_ is reduced to 100 ns when *ε*′ is used for amplitude correction, the energy resolution worsens only to 10.6% (*t*_Top_ = 10 ns). Thus, this temperature correction method can work well for a high count rate.

## 4. Discussion

The R^2^ values of the exponent-double exponent sum convolution models fitted to the average pulse at different temperatures were both greater than 0.9980, showing the best performance among the compared models. However, there was a certain deviation between the model and the real response. The high R^2^ values were partly due to the smoothness of the average pulses, and the fitting residual mainly reflected the model error. In fact, it can be seen in Figure 5 that there are certain differences between *ε* and *ε*′ for different trapezoidal parameters, which is caused by the model error. The actual scintillator luminescence process is very complicated [19,20,21], and is difficult to describe with mathematical functions. The pulse models used in practice are models that are simplified for practical purposes. In principle, a more complex model can be used to improve the accuracy of fitting, but it will increase the difficulty of fitting and subsequent deconvolution. From the practical point of view, the complexity and accuracy of the model should be considered comprehensively. In this paper, the exponent-double exponent sum convolution model was selected because of its moderate complexity. Although there was some deviation between the pulse model and the actual response, the consistency of pulse shapes after deconvolution and trapezoidal shaping at different temperatures shown in Figure 4 reflects the effectiveness of this method in pulse shape correction. Compared with previous temperature correction methods [5,6,7,8,9,10,11,12], this method can be used at a high count rate due to the correction of pulse shape and adjustable trapezoid width.

After correction using this method, the position drifts of the 662 keV peak at −20~50 °C were less than 3 keV (*ε*′). The sum spectra with the trapezoidal parameters of *t*_Width_ = 1000 ns and *t*_Top_ = 500 ns had a resolution of 6.91%@662 keV(*ε*′), which verifies the spectral stabilization effectiveness of the proposed method. It should be noted that this result was obtained under ideal conditions: firstly, the pulse model construction and spectral drift evaluation were all aimed at the 662 keV gamma rays; secondly, constant temperature conditions in the experiment ensured the consistency of the temperature in the whole NaI(Tl) crystal and PMT; thirdly, the temperature values used for calculating the model parameters were the same as those used for pulse processing, which prevented additional errors introduced by the model parameter estimation using temperature.

As shown in Figure 8, the energy resolution is related to the trapezoid width, *t*_Width_, and flat-top ratio, which is because trapezoid parameters affect the SNR of the trapezoid pulse. Figure 8 can provide a basic reference for selecting trapezoidal parameters at a specific count rate. The energy resolution deteriorates with the decrease in *t*_Width_, which can be attributed to the fact that the fewer data points are used to calculate the pulse amplitude information with the narrower trapezoidal width. Therefore, a relatively wide *t*_Width_ value should be adopted at a low count rate, and a smaller *t*_Width_ value should be adopted with the increase in the count rate in order to reduce the pile-up effect of the trapezoidal pulse. When the *t*_Width_ is small, the energy resolution becomes better with the decrease in the flat-top ratio. Therefore, it is advisable to adopt a smaller flat-top ratio at a high count rate. When using *ε*′ for amplitude correction, even if the *t*_Width_ is reduced to 100 ns, an energy resolution of 10.60% can still be obtained by adjusting the flat-top ratio, which preliminarily verifies the effectiveness of this method at a high count rate. It should be noted that, although the pulse pile-up effect can be reduced by using a smaller *t*_Width_ at a high count rate, the statistical fluctuations inherent in the pulse signals cannot be eliminated, which is reflected in the random fluctuation in the baseline on the right side next to the trapezoidal pulse in Figure 4c,d, and it will reduce the SNR of the trapezoid pulse. Therefore, the actual energy resolution at a high count rate will be worse than that shown in Figure 8.

In this paper, it was assumed that the pulse shape of NaI(Tl) should be independent of energy, and only one energy point of ^137^Cs 662 keV was verified. The consistency of the Compton edge in the energy spectra at different temperatures in Figure 6d can provide partial verification for the validity of the spectrum stabilization at other energy points. Due to the nonlinear energy response of the NaI(Tl) crystal [20,21], the hypothesis that the pulse shape is independent of energy cannot be perfectly confirmed in practice, so the effectiveness of this method with other energy points needs to be further verified.

In addition, in the amplitude correction step, only amplitude changes of the trapezoidal pulse caused by the NaI(Tl) response and temperature effect of the PMT gain were considered. The gain changes caused by the magnetic effect and count rate effect of the PMT still need to be stabilized by a magnetic shielding and voltage divider circuit design. Moreover, the amplitude correction step is independent of the steps of deconvolution and trapezoidal shaping. Therefore, the amplitude correction step can also be replaced by traditional spectral stabilization methods.

Finally, it should be noted that the model parameters obtained in this paper were derived from the crystal and PMT used. Since the pulse shape is affected to some extent by the crystal size, shape and the PMT response, it is best to refit the model parameters for different detector configurations including the crystal and PMT. If the detectors are of different configurations, it is suggested that they are fitted separately to obtain their own model parameters. For a particular detector, when the model parameters are obtained, the parameters are fixed and do not need to be changed for subsequent use. In the practical application, a pulse model can be applied to a batch of products. Of cause, strictly speaking, even if the detectors are of the same configuration, the pulse shapes may also differ slightly. Fitting model parameters individually for each detector can improve the accuracy of correction, but it increases the workload and seems generally unnecessary. In general, the correction error is only slightly increased when the model obtained from a single detector is applied to the whole batch of detectors. Additionally, in the process of fitting the pulse model, if several detectors could be randomly selected from a batch of products and the mean value of the average pulse of the detectors could be used to fit the model, the generalization of the model would be increased.

## 5. Conclusions

This paper proposed a temperature correction method for NaI(Tl) spectrometers, which is divided into three steps: deconvolution, trapezoidal shaping and amplitude correction. This method can be used to shape the pulses at different temperatures into regular trapezoidal pulses with the same *t*_Width_ and *t*_Top_, and the amplitude *H*_T_ of the trapezoidal pulse can be corrected to be equal to the pulse area, *A*_0_, at the reference temperature (20 °C). Compared to the existing spectrum stabilization methods based on adjusting the PMT’s high voltage or amplifier gain, the proposed method process scintillation pulses rather than the energy spectrum. This method can be used for field applications, including situations in which there is a high counting rate. Through adjusting the trapezoidal parameters, an acceptable energy resolution can be obtained. The results show that the proposed method can effectively correct the temperature response of NaI(Tl) detectors, and the position of the 662 keV peak changes by less than 3 keV with a temperature ranging from −20 °C to 50 °C. The trapezoidal parameters can affect the final energy resolution, but even when the trapezoidal pulse width is reduced to 100 ns, an energy resolution of 10.60% can still be ensured.

In this paper, the temperature values of the thermostatic chamber are directly used in the deconvolution operation. In practical applications, the temperature can be obtained using a temperature sensor or estimated via the method of pulse fitting or pulse shape parameter. It should be noted that the correction in this paper is only for the temperature effect of the scintillator and the PMT. Other factors affecting PMT gain and the instability of a high voltage and amplifier gain are not considered. These problems can be easily solved by replacing the amplitude correction step in this method with the traditional spectrum stabilization technique.

Although a NaI(Tl) detector is used in this paper, this method can be applied to other scintillation detectors, and the trapezoidal filter can also be replaced by an optimum filter to achieve a better energy resolution. Although the off-line pulse processing method is used in this paper, we are going to implement it in FPGA in the future.

## Figures and Tables

**Table 1:** Parameter fit results of exponent-double exponent sum convolution model.

Temperature/°C	*A*	*τ*_0_/ns	*τ*_1_/ns	*τ*_2_/ns	*φ*	*t*_0_/ns
−20	3.382 × 10^5^	9.784	160.8	842.9	0.2682	933.3
−10	3.302 × 10^5^	9.343	218.8	603.0	0.3202	933.3
0	3.194 × 10^5^	11.67	271.2	404.9	0.3778	937.4
10	3.059 × 10^5^	18.05	281.1	281.3	0.8297	930.6
20	2.935 × 10^5^	23.05	236.2	236.4	0.8120	929.4
30	2.785 × 10^5^	25.34	199.7	200.0	0.9993	929.3
40	2.625 × 10^5^	25.24	174.0	200.0	1.000	929.4
50	2.453 × 10^5^	23.93	155.9	200.0	1.000	929.7

**Table 2:** Fit results of *ε* and *ε*′ for different trapezoidal parameters.

		*a*_0_	*a*_1_	*a*_2_	R^2^
*ε*		−1.537 × 10^−5^	−4.398 × 10^−3^	1.095	0.9993
*ε*′	*t*_Width_ = 500 ns, *t*_Top_ = 100 ns	−2.928 × 10^−5^	−3.335 × 10^−3^	1.068	0.9997
	*t*_Width_ = 500 ns, *t*_Top_ = 250 ns	−4.033 × 10^−5^	−2.900 × 10^−3^	1.078	0.9993
	*t*_Width_ = 1000 ns, *t*_Top_ = 200 ns	−3.214 × 10^−5^	−3.345 × 10^−3^	1.077	0.9998
	*t*_Width_ = 1000 ns, *t*_Top_ = 500 ns	−2.861 × 10^−5^	−3.563 × 10^−3^	1.078	0.9998

## Data Availability

The data that support the findings of this study are available from the corresponding author upon reasonable request.
